# Handle shape influences system usability in telemanipulation

**DOI:** 10.3389/frobt.2024.1457926

**Published:** 2024-11-22

**Authors:** Esther I. Zoller, Sibylle von Ballmoos, Nicolas Gerig, Philippe C. Cattin, Georg Rauter

**Affiliations:** ^1^ Bio-Inspired RObots for MEDicine-Laboratory, Department of Biomedical Engineering, University of Basel, Allschwil, Switzerland; ^2^ Center for Medical Image Analysis and Navigation, Department of Biomedical Engineering, University of Basel, Allschwil, Switzerland

**Keywords:** ergonomics, handle, human factors, telemanipulation, usability

## Abstract

**Introduction:**

Ergonomic issues are widespread among surgeons performing teleoperated robotic surgery. As the ergonomics of a teleoperation system depends on the controller handle, it needs to be designed wisely. While the importance of the controller handle in robot-assisted telemanipulation has been highlighted previously, most existing work on the usability of a human-robot system for surgery was of qualitative nature or did not focus on surgery-specific tasks.

**Methods:**

We investigated the influence of nine different grasp-type telemanipulator handles on the usability of a lambda.6 haptic input device for a virtual six degrees of freedom peg-in-hole task. User performance with different handles was assessed through four usability metrics: i) task completion time, ii) dimensionless jerk, iii) collision forces, and iv) perceived workload. We compared these usability results with those of a prior study examining only the functional rotational workspace of the same human-robot system.

**Results:**

The linear mixed-effect model (LMM) analysis showed that all four usability metrics were dependent on the telemanipulator handle. Moreover, the LMM analysis showed an additional contribution of the hole accessibility to the usability of the human-robot system.

**Discussion:**

In case contact forces between the follower end-effector and its surroundings are not critical, the *fixed-hook*-grasp handle showed the best results out of the nine tested handles. In case low contact forces are crucial, the *tripod*-grasp handle was most suitable. It can thus be deduced that different grasp-type telemanipulator handles affect system usability for a surgery-related, teleoperated six degrees of freedom placement task. Also, maximizing the functional rotational workspace can positively affect system usability.

## 1 Introduction

Robotic technology is extensively used in surgical procedures (Tavakoli et al., 2008) in the form of teleoperated and image-guided systems (Hoeckelmann et al., 2015). For example, in 2023, over 2.2 million procedures were performed worldwide with da Vinci systems only (Intuitive Surgical, 2023), representing more than a 100% increase over the last 5 years (Intuitive Surgical, 2018). During teleoperated procedures, the surgeon operates a human-machine interface (the controller) that controls a remote surgical robot (the follower) (Tavakoli et al., 2008). The spatial separation of the surgeon from the operating instrument allows for processing the controller’s motions before transferring them to the follower. This results in several benefits of teleoperated surgery over traditional surgery, including tremor filtering, motion scaling, increased dexterity, and preventing the instrument from entering previously defined forbidden regions (Okamura, 2004; Park et al., 2001; Prasad et al., 2004; Tavakoli et al., 2008). However, these teleoperation systems also pose several challenges. Much effort has been put into solving technical challenges, such as overcoming the limited workspace of controllers (Conti and Khatib, 2005) or the lack of haptic feedback (Enayati et al., 2016; Okamura, 2004).

However, surgeon safety and comfort have received little attention in minimally invasive surgery (Catanzarite et al., 2018), such that it is not surprising that ergonomic issues are widespread among surgeons performing teleoperated robotic surgery (Armijo et al., 2019; Catanzarite et al., 2018; Craven et al., 2013; Morton and Stewart, 2022). The ideal arm position for working with surgical instruments has been described as follows (Matern and Waller, 1999; Matern, 2009): at the shoulder, the upper arm should be slightly abducted, retroverted, and rotated inward. The arm should be flexed 90°–120° at the elbow and the forearm in its natural position between pro- and supination. The hand should be in its natural position, with the wrist slightly extended and the fingers slightly bent. In accordance with this, the rapid upper limb assessment (RULA) survey for the ergonomic assessment of workplaces rates an upper arm position of 20° flexion to 20° extension with no elevation or abduction, a lower arm position of 60°–100° flexion and a neutral wrist position the most ergonomic (McAtamney and Corlett, 1993). The instrument handles influence the hand position of the user (Matern, 2009) and were found a possible target for ergonomic improvement of teleoperated robotic surgical systems (Morton and Stewart, 2022).

Important aspects of ergonomics are both human wellbeing and overall system performance (International Organization for Standardization, 2016). Overall system performance is a broad term and challenging to define and quantify. We considered system usability an essential aspect of overall system performance suitable for quantitative measurements. The ISO 9241–210 standard on “human-centred design for interactive systems” defines usability as the “extent to which a system […] can be used by specified users to achieve specified goals with effectiveness, efficiency and satisfaction in a specified context of use” (International Organization for Standardization, 2010). We are convinced that a telemanipulator handle influences the wellbeing of the human operating the robotic system and the usability of the human-robot system.

Already 30 years ago, Bejczy concluded, based on a subjective analysis, that for adequate control of six degrees of freedom (DoF), the telemanipulator handle “must be held firmly with at least two fingers and the heel of the hand at all times” (Bejczy, 1992). A newer survey on different surgical instrument handles (robotic and non-robotic) among surgeons showed that subjective handle preference was strongly influenced by the surgeons’ background and experience (Santos-Carreras et al., 2012). Yet, a study comparing different telemanipulators for teleoperation in earthwork highlighted the importance of considering factors for selecting the telemanipulator handle beyond user familiarity, such as telemanipulator ergonomics and task compatibility (Saunier et al., 2024). For robot-assisted surgery, two studies assessing both quantitative and qualitative performance metrics of different haptic telemanipulators highlighted the importance of the hand-controller (Baghdadi et al., 2020; Zareinia et al., 2015). However, in those studies both the mechanics and the handle of the compared telemanipulators differed. Hence, it remains unclear what performance differences emerge from the handle. It is thus not surprising that the authors themselves concluded that future work should focus on evaluating the performance of different handles with the same haptic telemanipulator. Our previous studies assessing the influence of the handle only on the usability of a human-robot system have focused on workspace exploring tasks (Zoller et al., 2019a; 2020). While the importance of the accessibility of a large workspace is undebatable during robot-assisted surgery, there are unarguably more representative tasks to assess the usability of a human-robot system for surgery. Thus, it can be concluded that previous work on telemanipulator handles was mostly of qualitative nature, did not focus on surgery-specific tasks, or stated the necessity of further studies comparing different handles with the same telemanipulator.

This study aimed to investigate the influence of different telemanipulator handles on the usability of a human-robot system. For this, we exemplarily chose a virtual six DoF peg-in-hole task, as many steps in a robotic surgery require the accurate placement of tools, which can be simulated by a six DoF peg-in-hole task. Also, peg-in-hole tasks have previously been used to assess user performance with robot-assisted surgical systems (Baghdadi et al., 2020; Okamura et al., 2011; Zareinia et al., 2015). In addition, we also investigated if system usability for a peg-in-hole task correlates with the human-robot system functional rotational workspace, i.e., the intersection of the mechanical rotational workspace of the telemanipulator device and the anatomical rotational workspace of the operator’s hand and wrist with a given grasp type and a weight-supported arm posture (Zoller et al., 2020). Such a correlation would underline the importance of maximizing the functional rotational workspace to improve telemanipulation with human-robot systems. Therefore, in this study the following hypotheses were tested:

Hypothesis 1 The telemanipulator handle influences system usability for a virtual peg-in-hole task.

Hypothesis 2 System usability for a virtual peg-in-hole task depends on the functional rotational workspace of the human-robot system.

## 2 Materials and methods

### 2.1 Input system

**FIGURE 1:**
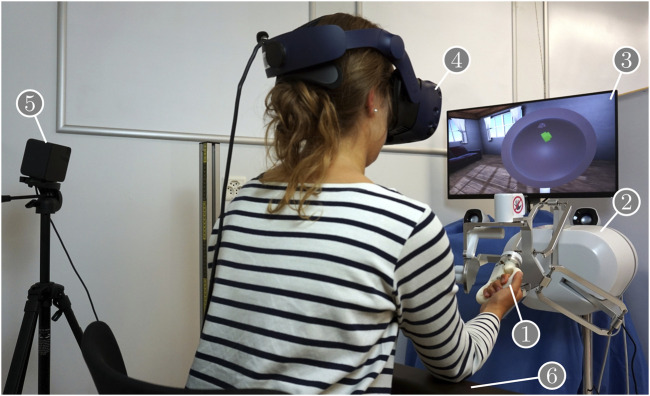
Experimental setup: the user interacts through the test handle ① mounted on the haptic input device ②‚ with a virtual environment ③. The virtual environment is visually perceived through the head-mounted display ④, which is tracked by a lighthouse system⑤. An armrest ⑥ allows support of the user’s forearm.

**FIGURE 2:**
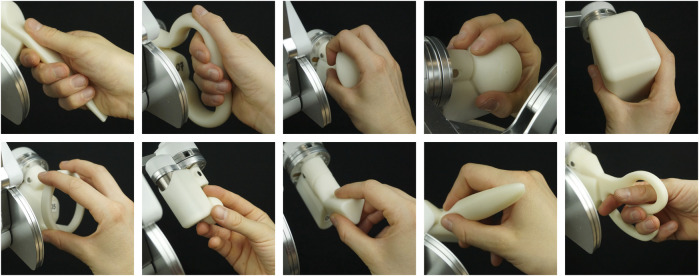
All nine test handles and the additional training handle used in this study are depicted. The top row depicts the five power-grip handles that are held with the following grasp types (left to right): adducted thumb, fixed hook, power disk, power sphere, and parallel extension. The bottom row depicts the four precision-grip handles that are held with the following grasp types: precision disk, quadpod, tripod, and writing tripod. At the bottom right, the training handle to be held with a distal grasp type is shown.

In this study, the usability of a haptic input device with different telemanipulator handles was assessed. The assessed haptic input device was a customized, handleless, six DoF lambda.6 (Force Dimension, Nyon, Switzerland), which was designed for right-handed use (see Figure 1). A detailed workspace description and illustrations for this device can be found in Zoller et al. (2020). An adapter at the end-effector of the lambda.6 device allowed for easy mounting of different handles. In this study, we used nine different handles (see Figure 2), each designed to be grasped with a different grasp type adopted from Feix et al. (2016). These were the same nine handles for which we have previously assessed the functional rotational workspace (Zoller et al., 2019a). We have developed these handles specifically for teleoperation tasks with the lambda.6 haptic input device (Zoller et al., 2019b). The computer-aided design (CAD) models of the handles used in this study are available online: https://doi.org/10.21227/8xhk-dv15.

### 2.2 Peg-in-hole scenario

To assess the usability of the input system, we designed a virtual reality (VR) scenario, which was displayed to the user on an HTC Vive Pro head-mounted display (HMD) tracked by a Lighthouse 1.0 system (both HTC, New Taipei City, Taiwan) (see Figure 1). The scenario consisted of a peg-in-hole task, in which a peg had to be inserted in different holes with different pitch/yaw/roll orientations displayed on the inside of a hemisphere. The scenario comprised two custom applications: a VR application used for visualization only and a C++ application for haptic rendering, control of the lambda.6 device, running the logic to complete the task, progressing through the study protocol, and data recording. The VR application was created in Unity (Unity Technologies, San Francisco, CA, United States), while the C++ application was created using CHAI3D (Conti et al., 2003) and the Force Dimension SDK (version 3.7.3.3210). The two applications communicated over network sockets to synchronize the relevant objects between the haptic and visual scenes and to exchange information about the VR scenario’s current state. Both applications were run as fast as possible, i.e., with target visual and haptic update rates of 60 Hz and 4,000 Hz, respectively. However, the actual update rates varied based on logged data. The measured visual and haptic update rates were 51 Hz ± 16 Hz and 2,700 Hz ± 1,087 Hz (mean ± std), respectively. Both the HMD and the haptic device were controlled by the same computer (HP Z640 Workstation, Intel Xeon E5-2630 CPU @ 2.2 GHz, Nvidia GTX 1080 GPU, 16 GB RAM, Windows 10).

The peg was shaped like a T-beam with a height, width, and depth of 37.5 mm each. The hemisphere had an outer radius of 200.25 mm and a thickness of 40.5 mm. For each trial, a hole with a specific pitch/yaw/roll orientation was displayed on the hemisphere. The hemisphere holes were slightly bigger than the peg, allowing for a rotational tolerance of 6 ° around each axis before collision if the peg position was perfectly centered in the hole. The center of each hole was at a distance of 180 mm from the sphere center. As the pose of the lambda.6 end-effector controlled the pose of the peg through direct mapping, the chosen dimensions allowed the user to perform the peg-in-hole task without any indexing. The visual scenario was displayed with a vertical shift of 0.3 m to enable the user adopting a more upright posture when performing the task. Thus, the hemisphere was visually displayed at a higher spatial location than perceived haptically. In pilot testing, we preferred the version including the vertical shift due to a subjectively perceived increase in visual 3D perception and scenery overview. While users need to adapt to such a positional visuomotor misalignment, they seem capable of compensating for it without any decrease in manipulation performance (Groen and Werkhoven, 1998). Also, for better visualization, a scaling factor of two was introduced between the physical environment and the virtual scene, enlarging the appearance of the hemisphere in the virtual workspace. Since our users needed to adapt and compensate for the visuomotor misalignment due to the visual shift, we have not considered this additional scaling to introduce perceivable complexity to the task.

**FIGURE 3:**
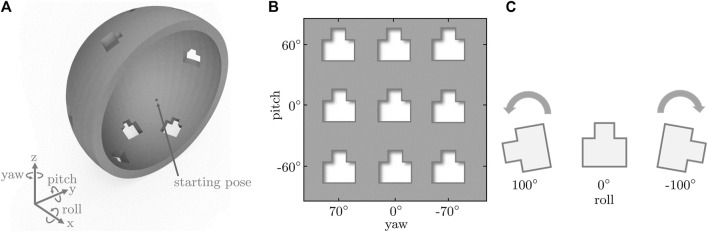
**(A)** Hemisphere with holes at the nine tested pitch/yaw orientations. **(B)** 2D map visualizing the different pitch/yaw orientations for the holes. **(C)** The three different roll orientations tested at each pitch/yaw orientation.

A total of 27 different holes with different pitch/yaw/roll orientations were displayed on the hemisphere one at a time (see Figure 3). We used the same pitch/yaw/roll orientations that we have previously used in the functional roll workspace assessments (Zoller et al., 2019a) to allow better relating the results of this usability study with previous findings about the functional rotational workspace of those nine tested grasp-type handles.

During the peg-in-hole task, the haptic device with the attached telemanipulation handles was controlled with gravity compensation and used by the participant to interact with the virtual environment displayed through the HMD. The VR scenario required the user to move the virtual peg to the hemisphere center in a neutral pitch/yaw/roll orientation before every peg insertion attempt to ensure a consistent starting pose for all peg insertion attempts. A ghost peg indicating the correct starting pose was visualized to facilitate maneuvering the peg to the correct pose. Also, a small magnetic force and torque helped the user position and orient the peg correctly as soon as they were close to the starting pose. In addition, linear and angular tolerances of 0.5 mm and 0.1 rad (5.73^◦^), respectively, were introduced for the starting pose. The user was required to hold the peg in the correct starting pose for 1.5 s to initiate the peg-in-hole task. During this period, the pose of the subsequent hole was displayed to the participant. The start of the task was then indicated with a sound, and the magnetic force and torque application was inactivated. During each peg insertion attempt, the user perceived haptic feedback in form of collision forces and torques when the peg collided with the hemisphere. Also, to protect the haptic device from any damage by the user, virtual haptic walls were implemented close to the mechanical joint limits of the device and rendered to the participants when they came close to the workspace boundaries of the haptic device. Each peg insertion attempt ended either with the peg insertion into the corresponding hole or after a trial duration of 30 s. A peg was considered inside a hole if its center was located at the center of the hole with a distance tolerance of 0.3 mm. At the end of each peg insertion attempt, another sound was played and the ghost peg at the starting pose was displayed to the user again. The user then had to move the handle back to the starting pose to align the peg with the ghost peg for the next peg-in-hole attempt. One scenario consisted of 27 peg insertion attempts into 27 holes with different pitch/yaw/roll orientations. During each peg insertion attempt, the C++ application recorded the position and orientation of both the lambda.6 end-effector and the virtual peg, as well as the forces and torques sent to the haptic device. The total attempt duration and the final positional and rotational errors of the peg with respect to its target pose in the hole were also recorded.

### 2.3 Usability metrics

The ISO 9241–210 standard on “human-centred design for interactive systems” mentions three aspects of system usability: effectiveness, efficiency, and satisfaction (International Organization for Standardization, 2010). The same standard defines effectiveness as “accuracy and completeness with which users achieve specified goals,” efficiency as “resources expended in relation to the accuracy and completeness with which users achieve goals,” and satisfaction as “freedom from discomfort and positive attitudes towards the use of the product.” In the ISO 2941–940 standard on the “evaluation of tactile and haptic interactions,” the expended resources defining the efficiency are further specified to include time, human effort, costs, and materials (International Organization for Standardization, 2017). The same standard states that to measure the user’s satisfaction with the system, one should consider their physical, cognitive, and emotional response. In order to cover all aspects of system usability, we investigated four different usability metrics: i) task completion time, ii) dimensionless jerk, iii) collision forces, and iv) perceived workload. The collision forces and dimensionless jerk represent the accuracy with which the users fulfilled the peg-in-hole tasks. The task completion time was used as an indirect measure of task completeness, as any task completion time <30 s represents a completed task, while a task completion time ≥ 30 s represents a failed task. Both the task completion time and the perceived workload cover the efficiency of using the human-robot system. The perceived workload was also used to measure the user’s satisfaction with the system.

### 2.4 Design and procedure

This study had a cross-over (within-subjects) design. Prior to the system usability assessment, the participant’s capability for stereo vision was tested using the Lang Stereotest I, as described by Brown et al. (2001). Furthermore, the participant’s hand size was assessed using a glove size chart. Then, the experimenter showed the participant the process of a peg-in-hole scenario with the training handle. During this process, the experimenter emphasized that each peg insertion should be carried out as fast as possible, but to protect the haptic device, calm, controlled movements would be preferred over fast, jerky movements. Also, the participant was reminded to respect the workspace limits of the haptic device rendered to the participant by virtual walls. Finally, the experimenter explained that each peg insertion attempt would end after 30 s but that they should keep trying to insert the peg until an attempt is either successful or aborted. For the peg-in-hole scenarios, the participant was seated in front of the lambda.6 haptic device, with an armrest supporting an ergonomic arm posture at rest. An instructional video was displayed to the participant on a computer screen, depicting how the tested handle should be grasped in the following peg-in-hole scenario. The participant was asked to grasp the handle correctly with the right hand and was corrected if necessary. Only the participant’s right hand was examined because both the device and the handles were designed for use with the right hand only. After verifying knowledge of the correct grasp, the participant let go of the handle, put the HMD on, re-grasped the handle, and completed the peg-in-hole scenario described in Section 2.2 with the goal of inserting the peg in each of the 27 holes as fast as possible. During the scenario, the experimenter continuously observed the grasp type of the participant and instructed the participant to restore the instructed grasp type when necessary.

Before switching to the next handle, the participant was asked to complete the NASA Raw Task Load Index (NASA-RTLX) questionnaire (Byers et al., 1989), assessing the perceived workload during the peg-in-hole scenario. Thus, one experiment included the instructional video, the peg-in-hole scenario, and the NASA-RTLX questionnaire.

For familiarization purposes, each participant conducted the experiment first with a training handle (*distal-type* grasp). Subsequently, the experiment was conducted nine times, once with each of the nine test handles. The order of the test handles was randomized using a 9th-order row-complete Latin square to account for learning and fatigue effects. Also, the order of the holes was randomized to be different for all nine scenarios assessing the test handles. In total, the experiment was conducted ten times per participant (once with the training handle and nine times with the test handles), resulting in a total duration of the experimental session of approximately 2 h per participant.

### 2.5 Participants

Twenty-eight healthy, right-handed participants (13 females, 23–62 years, mean age 31.3 years) volunteered to participate in this study in February 2020. All participants had normal or corrected to normal stereo vision and did not report any recent injury or other disorders of the right upper extremity. Twelve of the participants already participated in a former study of this study series (i.e., Zoller et al., 2019a and/or Zoller et al., 2020). Other than this, the participants did not have prior experience with haptic telemanipulators. The participants were recruited from the Department of Biomedical Engineering of the University of Basel (students and researchers) and the general public. The study was conducted according to the declaration of Helsinki, the law of Switzerland, and has been approved by the responsible ethics commission (EKNZ 2018-01992). Written informed consent was obtained from all participants. One participant decided to drop out of the study after suffering from a cramp during the experiments. This participant had been replaced with the 28th participant to guarantee that the total number of participants was a multiple of nine required for a Latin square randomization with nine conditions.

### 2.6 Data analysis

As described in Section 2.3, we assessed the four usability metrics task completion time, dimensionless jerk, collision forces, and perceived workload. The data from the training handle was not analyzed. While task completion time, dimensionless jerk, and collision forces were analyzed for each peg insertion attempt, the perceived workload was assessed once for each grasp-type handle per participant.

The task completion time for each peg insertion attempt was directly recorded by the C++ application.

For the dimensionless jerk, we used the dimensionless jerk measure (DJ) as proposed by Hogan and Sternad (2009) (Equation 1):

$$DJ=\int_{t_{1}}^{t_{N}}\dddot{x}{\left(t\right)}^{2}dt\;\frac{{\left(t_{N}-t_{1}\right)}^{3}}{v_{\mathrm{mean}}^{2}},$$ (1)

where DJ is the dimensionless jerk, i.e., the inverse of movement smoothness, x(t) the time-dependent position, $v_{\mathrm{mean}}$ the mean velocity, and $t_{N}-t_{1}$ the duration of the movement. We approximated DJ numerically because of the discretely measured position data of the lambda.6 end-effector (Equation 2):

$$DJ\approx \overset{N}{\underset{i=1}{\sum }}{\hat{j}}_{i}^{2}\;\left(t_{i}-t_{i-1}\right)\;\frac{{\left(t_{N}-t_{1}\right)}^{3}}{{\hat{v}}_{\mathrm{mean}}^{2}},$$ (2)

with $\hat{j_{i}}$ the smoothed movement jerk and ${\hat{v}}_{\mathrm{mean}}$ the mean of the smoothed movement velocity $\hat{v_{i}}$ . $\hat{j_{i}}$ and $\hat{v_{i}}$ were obtained by smoothing the discrete derivatives $j_{i}$ and $v_{i}$ with a moving average filter ( n=5 , equal weights). The discrete derivatives $j_{i}$ and $v_{i}$ were obtained by the five-point stencil on downsampled position data (100 Hz), i.e., the movement jerk $j_{i}$ was approximated using

$$j_{i}\approx \frac{x\left(t_{i}+2h\right)-2x\left(t_{i}+h\right)+2x\left(t_{i}-h\right)-x\left(t_{i}-2h\right)}{2h^{3}}$$ (3)

and the movement velocity $v_{i}$ was approximated using

$$\upsilon_{i}\approx \frac{-x\left(t_{i}+2h\right)+8x\left(t_{i}+h\right)-8x\left(t_{i}-h\right)+x\left(t_{i}-2h\right)}{12h},$$ (4)

where h=0.01s (Equations 3, 4 derived in Supplementary Material).

For the collision forces, the force data was also downsampled to 100 Hz and only time points at which the recorded force data was > 0 N were considered. We then calculated the mean magnitude of the collision forces sent to the haptic device for rendering for each peg insertion attempt.

The perceived workload was computed once for each handle per participant from the NASA-RTLX questionnaire. This questionnaire consisted of six questions (see Supplementary Material) that the participants answered by crossing one of twenty-one lines dividing a 100-point scale into 5-point increments. The perceived workload score is the mean of the six individual scales (Byers et al., 1989).

The data of the 81 peg insertion attempts ( 3×27 , handles × holes) of the participant that dropped out of the study were discarded. Of the remaining 6,561 peg insertion attempts ( 27×9 × 27, participants × handles × holes), 109 failed, i.e., the attempt was aborted after 30 s. The recorded data (task completion time, dimensionless jerk, and collision forces) were still used for data analysis. Additionally, we modified the data of seven peg insertion attempts before data evaluation due to minor technical or protocol issues. During five peg insertion attempts, a participant let go of the test handle. In these attempts, we set the task completion time to the maximum, i.e., 30 s, and ignored the dimensionless jerk and collision forces for the data analysis. The perceived workload scores for the corresponding handles were not modified. During one peg insertion attempt, no collision forces were rendered between the peg and the hemisphere. For this attempt, we ignored all quantitative metrics for the data analysis. Again, the perceived workload scores for the corresponding handle were not modified. Finally, during one peg insertion attempt, the recording of the haptic device end-effector and virtual peg data failed. For this attempt, no data was available for the dimensionless jerk and collision forces. The task completion time and perceived workload scores were not affected or modified.

The statistical analysis of the data was performed in R (version 3.6.3) using an alpha level of .05. We performed a linear mixed-effect model (LMM) analysis for each of the four different usability metrics using the lme4 library (Bates et al., 2015). LMMs were chosen since the participants were measured repeatedly and thus, the different data from one participant are not independent. Also, compared to repeated measures ANOVA, LMMs can better deal with missing data and allow modeling dependent variables with numeric (integer and floating point) factors on top of categorical factors. For task completion time, dimensionless jerk, and collision forces, the LMM analysis was performed on the level of each individual peg insertion attempt. The LMM analysis for perceived workload was performed on the level of each handle. All LMMs were fit with maximum likelihood estimation. For each usability metric y , the LMM analysis was started with a global intercept and a participant-specific random effect correcting for within-subject correlation (Equation 5):

$$y∼1+\left(1\;|\;participant\right)$$ (5)

To test our hypothesis that the telemanipulator handle influences system usability for a peg-in-hole task (Hypothesis 1), the models were extended by adding the hypothesized predicting variable *handle* (factor with nine levels) as a fixed effect (Equation 6):

$$y∼handle+\left(1\;|\;participant\right)$$ (6)

Likelihood ratio tests $\left(\chi^{2}\right)$ were used to test the extended models against their parents. Each extended model had to explain the observed data significantly better than its parent model, or it was rejected. For the resulting LMMs, the assumption of normally distributed residuals was visually inspected using a normal Q-Q plot. In case of a heavy-tailed residual distribution, we repeated the analysis with the log-transformed data of the respective usability metric. Also, the participant-specific random effect in the resulting LMMs was assessed using profiling likelihood. Finally, a pairwise comparison of the handles was performed using the Tukey honestly significant difference test and the resulting best-fitting LMMs.

To test the hypothesis that system usability is influenced by the functional rotational workspace of the human-robot system (Hypothesis 2), the models were further extended by adding the numeric variable *insideWristRoM* as a predictor [*insideWristRoM* from 0 to 9, data from Zoller et al. (2019a)]. This variable designates how many participants reached a specific hole orientation when only movements of the hand and wrist were allowed and is thus a measure of the accessibility of each hole orientation. Thus, two additional LMMs were fit (Equations 7, 8):

$$y∼handle+insideWristRoM+\left(1\;|\;participant\right)$$ (7)

$$y∼handle+insideWristRoM+handle\;:\;insideWristRoM+\left(1\;|\;participant\right),$$ (8)

where y designates the three quantitative usability metrics task completion time, dimensionless jerk, and collision forces, since hole-specific data is not available for the RTLX questionnaire. Again, the extended models were tested against their parent with likelihood ratio tests $\left(\chi^{2}\right)$ and rejected if they failed to explain the observed data significantly better than their parent. Also, the assumption of normally distributed residuals was visually inspected using a normal Q-Q plot for the resulting LMMs. In case of a heavy-tailed residual distribution, we repeated the analysis with the log-transformed data of the respective usability metric.

## 3 Results

LMM analysis of all quantitative usability metrics (task completion time, dimensionless jerk, and collision forces) resulted in heavy-tailed normal Q-Q plots, which violated the assumption for normally distributed residuals. Therefore, the LMM analysis was completed using the natural log transform of task completion time, dimensionless jerk, and collision forces as the dependent variables. The histograms of both the original and, where applicable, log-transformed data as well as the normal Q-Q plots of the resulting LMMs can be found in the Supplementary Material.

**FIGURE 4:**
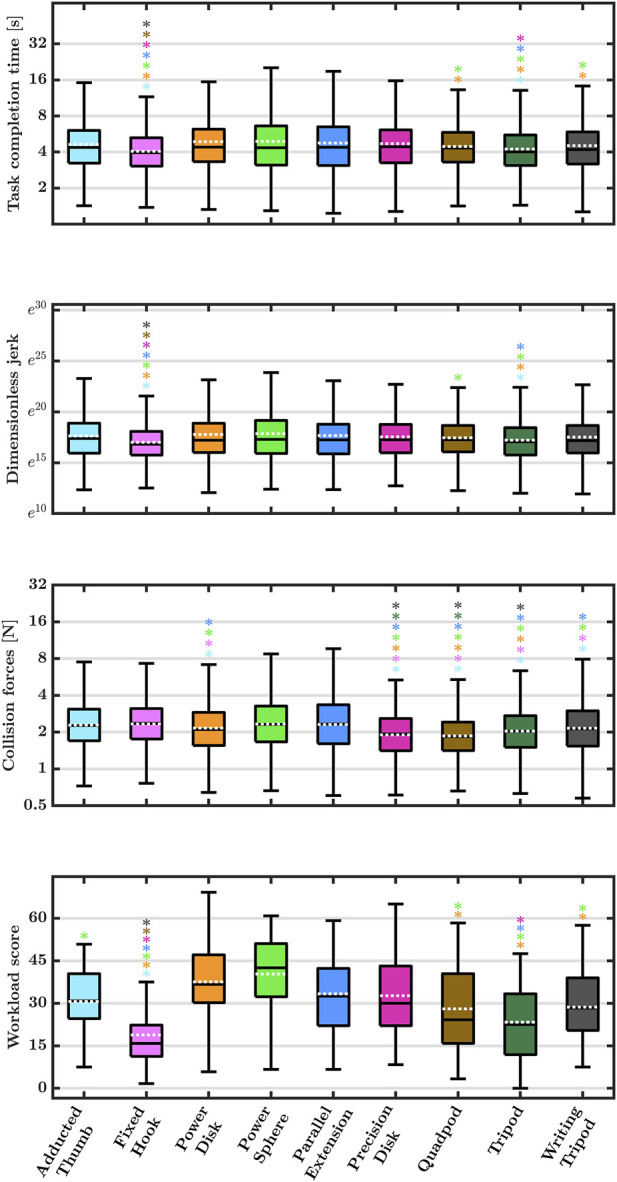
The task completion time, dimensionless jerk, collision forces (logarithmic scale), and the workload of the participants for the different grasp-type handles. All peg insertion attempts were considered for the task completion time, dimensionless jerk, and collision forces. The perceived workload was assessed once per participant and handle. Low values indicate fast task completion, high movement smoothness, gentle peg insertion, and low mental effort, respectively. The solid central line on each box indicates the median and the bottom and top edges of the boxes designate the 25th $\left(q_{1}\right)$ and 75th $\left(q_{3}\right)$ percentiles, respectively. The whiskers extend to the most extreme data points not considered outliers. Data points greater than $q_{3}+1.5\times \left(q_{3}-q_{1}\right)$ or less than $q_{1}-1.5\times \left(q_{3}-q_{1}\right)$ were considered outliers and are not visualized. The dashed white line on each box indicates the mean. Significant differences (p<.05) between the different handles are indicated with **∗** above the handles with which the participants completed the task faster, with smoother movements, lower collision forces, and with which the workload was perceived to be lower, respectively. The color of the asterisk indicates the grasp-type handle with which participants completed the task slower, with less smooth movements, higher collision forces, and with which the workload was perceived to be higher, respectively.

For all four usability metrics (task completion time, dimensionless jerk, collision forces, and perceived workload), the likelihood ratio test showed a significantly better model fit for the model with handle as a fixed effect (Equation 6) compared to the model with no fixed effect (Equation 5, p <.001 ). Figure 4 visualizes how the different grasp-type handles varied in the four usability metrics as well as the results of the pairwise comparisons with the Tukey honestly significant difference test. The numerical results of these pairwise comparisons are shown in Table 1.

**TABLE 1:** Tukey honestly significant difference *post hoc* test results for the pairwise handle comparisons and all four usability metrics.

Pairwise handle comparison	Task completion time			Dimensionless jerk			Collision forces			Workload score		
	z	p	Δ [s]	z	p	Δ [-]	z	p	Δ [N]	z	p	Δ [-]
**Adducted thumb**												
− **Fixed hook**	5.454	<.001	0.949	5.589	<.001	${2.118\cdot 10}^{9}$	−1.032	.983	−0.011	4.518	<.001	11.790
− **Power disk**	−2.166	.429	−0.778	−1.275	.939	$-5.578\cdot 10^{9}$	3.223	.034	0.087	−2.661	.163	−6.944
− **Power sphere**	−2.398	.285	−0.915	−1.995	.547	$-4.381\cdot 10^{9}$	−0.873	.994	−0.097	−3.714	.006	−9.691
− Parallel extension	−1.094	.975	−0.561	−0.345	1.000	$-3.752\cdot 10^{9}$	−0.156	1.000	−0.093	−1.041	.982	−2.716
− **Precision disk**	−0.611	1.000	−0.115	0.873	.994	$0.939\cdot 10^{9}$	10.179	<.001	0.504	−0.781	.997	−2.037
− **Quadpod**	1.648	.778	0.510	1.620	.794	$2.227\cdot 10^{9}$	11.156	<.001	0.498	0.994	.987	2.593
− **Tripod**	3.748	.006	0.697	3.705	.006	${2.028\cdot 10}^{9}$	6.338	<.001	0.278	2.791	.118	7.284
− **Writing Tripod**	1.025	.984	0.086	1.049	.981	$0.135\cdot 10^{9}$	3.189	.039	0.091	0.757	.998	1.975
**Fixed hook**												
− **Power disk**	−7.620	<.001	−1.727	−6.864	<.001	${-7.696\cdot 10}^{9}$	4.255	<.001	0.098	−7.179	<.001	−18.735
− **Power sphere**	−7.852	<.001	−1.864	−7.584	<.001	${-6.499\cdot 10}^{9}$	0.158	1.000	−0.086	−8.232	<.001	−21.481
− **Parallel extension**	−6.548	<.001	−1.510	−5.934	<.001	${-5.870\cdot 10}^{9}$	0.876	.994	−0.082	−5.559	<.001	−14.506
− **Precision disk**	−6.064	<.001	−1.063	−4.704	<.001	${-1.179\cdot 10}^{9}$	11.209	<.001	0.515	−5.299	<.001	−13.827
− **Quadpod**	−3.806	.004	−0.439	−3.967	.002	${0.109\cdot 10}^{9}$	12.187	<.001	0.510	−3.525	.012	−9.198
− **Tripod**	−1.707	.743	−0.252	−1.884	.625	$-0.090\cdot 10^{9}$	7.370	<.001	0.289	−1.727	.731	−4.506
− **Writing Tripod**	−4.430	<.001	−0.863	−4.540	<.001	${-1.983\cdot 10}^{9}$	4.220	<.001	0.102	−3.761	.005	−9.815
**Power disk**												
− **Power sphere**	−0.232	1.000	−0.137	−0.720	.999	$1.197\cdot 10^{9}$	−4.096	<.001	−0.184	−1.053	.981	−2.747
− **Parallel extension**	1.072	.978	0.217	0.930	.991	$1.826\cdot 10^{9}$	−3.378	.021	−0.180	1.620	.794	4.228
− **Precision disk**	1.554	.830	0.663	2.146	.442	$6.517\cdot 10^{9}$	6.963	<.001	0.417	1.881	.627	4.907
− **Quadpod**	3.814	.004	1.288	2.894	.090	$7.805\cdot 10^{9}$	7.934	<.001	0.411	3.655	.008	9.537
− **Tripod**	5.913	<.001	1.475	4.980	<.001	${7.606\cdot 10}^{9}$	3.116	.048	0.191	5.453	<.001	14.228
− **Writing Tripod**	3.190	.038	0.864	2.324	.327	$5.713\cdot 10^{9}$	−0.034	1.000	0.004	3.418	.019	8.920
**Power sphere**												
− Parallel extension	1.304	.930	0.354	1.650	.777	$0.629\cdot 10^{9}$	0.718	.999	0.004	2.673	.158	6.975
− **Precision disk**	1.786	.692	0.800	2.865	.098	$5.320\cdot 10^{9}$	11.051	<.001	0.601	2.933	.081	7.654
− **Quadpod**	4.046	.002	1.425	3.614	.009	${6.608\cdot 10}^{9}$	12.029	<.001	0.596	4.707	<.001	12.284
− **Tripod**	6.145	<.001	1.612	5.700	<.001	${6.409\cdot 10}^{9}$	7.212	<.001	0.375	6.505	<.001	16.975
− **Writing Tripod**	3.422	.018	1.001	3.044	.060	$4.516\cdot 10^{9}$	4.062	.001	0.188	4.471	<.001	11.667
**Parallel extension**												
− **Precision disk**	0.482	1.000	0.446	1.218	.953	$4.691\cdot 10^{9}$	10.335	<.001	0.597	0.260	1.000	0.679
− **Quadpod**	2.741	.134	1.071	1.964	.569	$5.979\cdot 10^{9}$	11.311	<.001	0.591	2.034	.519	5.309
− **Tripod**	4.841	<.001	1.258	4.050	.002	${5.780\cdot 10}^{9}$	6.494	<.001	0.371	3.832	.004	10.000
− **Writing Tripod**	2.118	.461	0.647	1.394	.901	$3.887\cdot 10^{9}$	3.344	.023	0.184	1.798	.684	4.691
**Precision disk**												
− Quadpod	2.259	.368	0.625	0.743	.998	$1.288\cdot 10^{9}$	0.956	.990	−0.005	1.774	.700	4.630
− **Tripod**	4.358	<.001	0.812	2.824	.108	$1.089\cdot 10^{9}$	−3.854	.004	−0.226	3.572	.011	9.321
− **Writing Tripod**	1.636	.785	0.201	0.173	1.000	$-0.804\cdot 10^{9}$	−6.998	<.001	−0.413	1.538	.838	4.012
**Quadpod**												
− **Tripod**	2.100	.473	0.187	2.084	.485	$-0.199\cdot 10^{9}$	−4.819	<.001	−0.220	1.798	.684	4.691
− **Writing Tripod**	−0.623	.999	−0.424	−0.571	1.000	$-2.092\cdot 10^{9}$	−7.968	<.001	−0.408	−0.237	1.000	−0.617
**Tripod**												
− **Writing Tripod**	−2.723	.140	−0.611	−2.656	.163	$-1.893\cdot 10^{9}$	−3.150	.043	−0.187	−2.034	.519	−5.309

Reported z- and *p*-values of the performed statistics are based on the log-transformed task completion times, dimensionless jerks, and collision forces; and raw workload scores. The raw, not log-transformed group mean differences Δ are reported additionally to provide an intuitive, physical unit difference. However, raw group mean differences have to be interpreted with care, because unlike the applied statistics these are not corrected for within-subject correlation.

z represents the statistics’ z-value and p its associated *p*-value. Handle comparisons with a *p*-value below the specified alpha level of .05 are printed in bold font. Δ represents the difference in the mean of each usability metric between the two compared handles. All our usability metrics denote an outcome where “less is better”, a positive delta therefore indicates that the second handle was superior, whereas a negative delta denotes that the first handle was superior.

For the three quantitative usability metrics (task completion time, dimensionless jerk, and collision forces), the likelihood ratio test showed a significantly better model fit for the model with both insideWristRoM and the handle as fixed effects (Equation 7) compared to the model with only the handle as a fixed effect (Equation 6, p <.001 ). In addition, the model that also included an interaction of the two fixed effects (Equation 8) showed a significantly better model fit than the model with the two fixed effects without an interaction (Equation 7, p <.001 ). For the task completion time, the dimensionless jerk, and the collision forces, a negative correlation with the fixed-forearm accessibility of the hole orientation was found for each handle (for details see Supplementary Material).

## 4 Discussion

Former work on usability-related metrics of human-robot systems for surgery focused on comparing different haptic devices (Baghdadi et al., 2020; Zareinia et al., 2015). Regarding the handles of surgical devices, previous work mainly focused on its design for an ergonomic work position, the possibility to trigger additional device functionality, a large functional workspace, or the handle comfort and precision (Bejczy, 1992; Matern and Waller, 1999; Matern, 2009; Santos-Carreras et al., 2012; Zoller et al., 2019a; 2020). Complementary to this, we focused on the quantitative usability assessment of one specific haptic device with different handles for a specific task mimicking the common surgical requirement to place a tool accurately.

Our results showed that all four usability measures were significantly influenced by the telemanipulator handle, i.e., our model with handle as a fixed effect (Equation 6) fit the data better than the model with no fixed effect (Equation 5). Thus, our first hypothesis that the telemanipulator handle influences system usability has been confirmed. The normal Q-Q plots of the resulting LMMs indicate that our data were not completely normally distributed (see Supplementary Material). Still, the chosen LMMs fit the majority of our data, and we thus decided against using more complex models as the chosen LMMs present a widespread statistical methodology allowing readers to easily understand and interpret the results.

One might argue that a single usability score combining the four usability metrics might allow for a better comparison of the different handles. Such a combination would require weighting the different metrics. We decided against such a combination because different real-life tasks would require different weightings. Low task completion time is always aimed for in surgical settings to minimize the anesthesia time and cost of operating room use. However, especially when interacting with delicate tissue such as nerves, low dimensionless jerk and collision forces indicate smoother and more gentle tissue interaction, potentially preventing tissue damage and thus improving surgical outcomes. This would be more important than shorter operating times in surgeries with delicate tissues nearby. Therefore, such weighting would be task-dependent, e.g., different for orthopedic vs. brain surgery.

Regarding the task completion time, the dimensionless jerk, and the perceived workload score, the *fixed-hook*-, *quadpod*-, *tripod*-, and *writing-tripod*-grasp handles showed the lowest mean values across all peg insertions. For the *fixed-hook*-, *quadpod*-, and *tripod*-grasp handles, these low mean values also resulted in significantly lower task completion time, dimensionless jerk, and perceived workload score compared to other handles. These results indicate that these handles were more suitable for the investigated peg-in-hole task than others, as they required less time, presented a lower workload, and resulted in smoother movements.

A different pattern was observed for the collision forces: here, the *precision-disk*-, *quadpod*-, *tripod*-, and *writing-tripod*-grasp handles showed the lowest mean values across all peg insertions. These low mean values also resulted in significantly lower collision forces compared to other handles. Collision forces are a measure of placement accuracy because they are proportional to the unwanted penetration depth of the peg into the virtual hole walls. As all four of the above handles are grasped with a precision grip with an abducted thumb (Feix et al., 2016), it can be assumed that for low collision forces in an accurate placement task, it is best to choose a precision grip with an abducted thumb. This is supported by previous findings where surgeons perceived handles whose primary function (instrument aperture) was controlled with a power grip as less accurate (Santos-Carreras et al., 2012).

The *fixed-hook*-grasp handle scored significantly better than all other handles except for the *tripod*-grasp handle in three of our four usability metrics (task completion time, dimensionless jerk, and perceived workload). This result is surprising as Santos-Carreras et al. (2012) concluded based on a qualitative handle evaluation that handles “which need to be grasped with the whole hand”, i.e., power-grip handles (Napier, 1956) such as the *fixed-hook*-grasp handle (Feix et al., 2016), are less intuitive. The sub-scale “mental demand” of the RTLX can be seen as a measure of intuitiveness (Hurtienne, 2011). A closer evaluation of the mental demand sub-scale revealed the lowest mean scores (16.3 of 100) for the *fixed-hook*- and *tripod*-grasp handles. Our results, therefore, do not support the conclusion that power-grip handles are, *per se*, less intuitive to use than precision grip handles.

The *fixed-hook*-grasp handle scored best in three of our four usability metrics (task completion time, dimensionless jerk, and perceived workload; see z -scores in Table 1) but showed relatively high collision forces. Such a handle might be best for the rough positioning of a tool or longer tasks where low contact forces are not crucial. Combining our findings with those of Santos-Carreras et al. (2012), using a *fixed-hook*-grasp handle with a seventh DoF that is controlled by the index finger and thumb only might be a good choice for tasks that require a seventh DoF, e.g., an instrument aperture. In such a handle, the *fixed-hook* grasp would provide the stability and comfort of the user, while the precision grip for the seventh DoF would provide the required precision. On the other hand, our findings suggest that a *tripod*-grasp handle would be the best choice for shorter tasks where low contact forces are crucial. Thus, we would assume that an openable *tripod*-grasp handle could also be a good choice for seven DoF tasks. However, we consider future quantitative investigations of the influence of telemanipulator handles on system usability for seven DoF tasks with known contact force requirements as highly relevant to improving teleoperation systems.

Including insideWristRoM as a fixed effect in our data fitting model (Equation 7) significantly improved the model. Thus, our second hypothesis that system usability for a peg-in-hole task depends on the functional rotational workspace of the human-robot system has also been confirmed. For every handle, the LMMs resulted in a negative correlation of all three quantitative usability metrics (task completion time, dimensionless jerk, and collision forces) with the fixed-forearm accessibility of the hole orientation. It can thus be concluded that the pegs were inserted faster, with smoother movements, and more gently in hole locations accessible with wrist motion only.

The only difference between the conditions in both this and our previous studies (Zoller et al., 2019a; Zoller et al., 2020) was the used handle requiring different grasp types. Therefore, our observed correlation between the usability metrics and the fixed-forearm accessibility of the rotational workspace indicates that the user’s initial hand position, i.e., the grasp type, alone influences telemanipulator usability. This indicates that maximizing the fixed forearm rotational workspace, which we aimed for in previous work (Zoller et al., 2020), can already be used to improve the usability of a telemanipulator handle. We consider this an important finding because the functional rotational workspace can be evaluated with much simpler setups than the usability, i.e., with less elaborate virtual environments and without haptic feedback. Thus, simpler and task-independent experiments to improve the rotational workspace of a handle could already be used to improve system usability.

Robotic surgery is more fatiguing for the shoulder and neck than laparoscopic surgery (Armijo et al., 2019). Besides the positive correlation with usability, maximizing the fixed forearm rotational workspace reduces required shoulder and elbow movements. That could reduce the fatiguing shoulder movements during telemanipulation, resulting in further ergonomic benefits. However, reduced muscle fatigue cannot be deduced from our data and would require a different study protocol. In addition, muscle fatigue is likely task-dependent, e.g., specific to posture during and the duration of the teleoperation task.

In this study, we assessed a simulated peg-in-hole-task using a virtual end-effector instead of a physical follower device controlled by the telemanipulator. While surgical instrument handles can be studied in virtual reality settings (Matern et al., 2005), the performance will always depend on certain properties of the virtual environment (Gillespie, 2005). In our case, the resolution and contrast of the HMD or the haptic force and torque rendering might have influenced performance. However, the limitations of the virtual environment were identical for the trials with all handles. Therefore, we would not expect them to have affected the differences found between the handles. Nevertheless, in a real teleoperation setting, task performance and user experience would be influenced by additional challenges on the follower side, such as delays, limitations of control, stability issues, and precision limitations in position, velocities, and forces. Also, physical phenomena such as friction and slipping are difficult to simulate accurately and might have an additional effect on task performance and user experience. Because different handles might influence how users can cope with these challenges, further studies with a real follower device are needed to verify our findings on the influence of a telemanipulator handle on system usability.

Also, certain characteristics of the user influence performance (Gillespie, 2005). We had participants with very diverse levels of experience interacting with virtual environments. In our opinion, this is rather a strength than a limitation because we assume that surgeons’ experience interacting with virtual environments varies largely from none to extensive. Thus, the experience of our study population in interacting with virtual environments supposedly mirrors that of the surgeon population, i.e., the end users of surgical telemanipulators. The age distribution of the participants in this study did not represent the population likely to be involved in teleoperated surgery because no participant was in the age range between 35 and 50 years. However, we do not expect findings contrary to this study for participants in this age group.

Future studies could validate our findings by replacing our virtual environment with a real follower and a real six DoF peg-in-hole task. For this, any robot suitable for robotic surgery, such as the ones described by Alip et al. (2022), could be used if it is controlled by a haptic device that allows mounting different grasp-type handles. However, in our understanding, usability and performance are strongly dependent on the investigated task, therefore, generalization of findings is often questionable. As surgical procedures are more complex than a peg-in-hole task, we highly suggest investigations on the individual systems and tasks at hand. For this, a specific seven DoF task, such as a sewing task, could be investigated by comparing system usability with two different seven DoF handles, a *fixed-hook*-grasp handle with a seventh DoF that is controlled by the index finger and thumb and an openable *tripod*-grasp handle. Nevertheless, given that we found significant effects in a simplified sample task, it can be assumed that these effects become even more pronounced with more complex tasks. We are thus convinced that our results provide two helpful insights applicable to many cases: First, simply maximizing the fixed-forearm workspace may already improve usability independent of the specific task at hand. Secondly, different handles indeed make a difference for peg-in-hole-like tasks, and *fixed-hook*- and *tripod*-grasp handles are promising.

## 5 Conclusion

To our knowledge, this study is the first to assess the usability of different handles with the same haptic telemanipulator. In our investigated virtual peg-in-hole task, different telemanipulator handles significantly affected system usability in all four measured performance metrics task completion time, dimensionless jerk, collision forces, and perceived workload. Best performances were achieved with the *fixed-hook*- and *tripod*-grasp handles. Our results indicate that precision grip handles with an abducted thumb might be best for accurate placement tasks where low collision forces are crucial. In contrast, *fixed-hook*-grasp handles might be a good choice for the rough positioning of a tool or tasks of long duration, given that low contact forces are not crucial.

Additionally, we have shown a correlation between the fixed-forearm accessibility of the rotational workspace with the three measured quantitative performance metrics task completion time, dimensionless jerk, and collision forces. Fixed-forearm accessibility is task-independent and can be improved with simple means (Zoller et al., 2020). Our observed correlation indicates that maximizing fixed-forearm accessibility can positively affect system usability and that the user’s initial hand position alone is already influencing telemanipulator usability. Nevertheless, to be applicable to surgical robotics, our findings must be validated in a real teleoperation setting investigating specific surgical tasks.

## Data Availability

The raw data supporting the conclusions of this article will be made available by the authors, without undue reservation.
