# Supplementary material for: Handle shape influences system usability in telemanipulation
Source: Front Robot AI. 2024 Nov 22;11:1457926. doi: 10.3389/frobt.2024.1457926 (PMC11620994; doi:10.3389/frobt.2024.1457926)
Supplement: Supplementary file 1 [file DataSheet1.pdf]

Supplementary Material

1 NUMERICAL APPROXIMATION OF DERIVATIVES

The numerical approximation of the derivatives used to calculate the dimensionless jerk can be derived from the Taylor series of $x(t \pm h)$ and $x(t \pm 2h)$ up to the third derivative of $x(t)$. At points $t + h$ and $t - h$, we have

$$x(t \pm h) \approx x(t) \pm h\dot{x}(t) + \frac{h^2}{2}\ddot{x}(t) \pm \frac{h^3}{6}\dddot{x}(t). \tag{1$\pm$}$$

Evaluating Eq. $(1+)$ – Eq. $(1-)$ gives

$$x(t + h) - x(t - h) \approx 2h\dot{x}(t) + \frac{h^3}{3}\dddot{x}(t). \tag{1}$$

Similarly, at points $t + 2h$ and $t - 2h$, we have

$$x(t \pm 2h) \approx x(t) \pm 2h\dot{x}(t) + \frac{4h^2}{2}\ddot{x}(t) \pm \frac{8h^3}{6}\dddot{x}(t). \tag{2$\pm$}$$

Eq. $(2+)$ – Eq. $(2-)$ gives

$$x(t + 2h) - x(t - 2h) \approx 4h\dot{x}(t) + \frac{8h^3}{3}\dddot{x}(t). \tag{2}$$

Combining Eq. 1 and Eq. 2 results in the numerical approximation of the first derivative

$$\dot{x}(t) \approx \frac{8x(t + h) - 8x(t - h) - x(t + 2h) + x(t - 2h)}{12h} \tag{3}$$

and that of the third derivative

$$\dddot{x}(t) \approx \frac{x(t + 2h) - x(t - 2h) - 2x(t + h) + 2x(t - h)}{2h^3}. \tag{4}$$

2 NASA-RTLX QUESTIONNAIRE

The perceived workload was computed from the NASA-RTLX questionnaire. This questionnaire consisted of the following six questions that were answered by the participants by crossing one of twenty-one lines dividing a 100-point scale into 5-point increments:

1. Mental Demand – How mentally demanding was the task?
2. Physical Demand – How physically demanding was the task?
3. Temporal Demand – How hurried or rushed was the pace of the task?
4. Performance – How successful were you in accomplishing what you were asked to do?
5. Effort – How hard did you have to work to accomplish your level of performance?
6. Frustration – How insecure, discouraged, irritated, stressed, and annoyed were you?

The perceived workload score is the mean of the six individual scales Byers et al. (1989).

3 DETAILED RESULTS

Raw data and resulting linear mixed models (LMMs) including an interaction of the two fixed effects *handle* and *insideWristRoM* (Eq. 8 in the main paper) are displayed for each of the three quantitative usability metrics, separated by grasp-type handles, in Figs. S1 to S3.

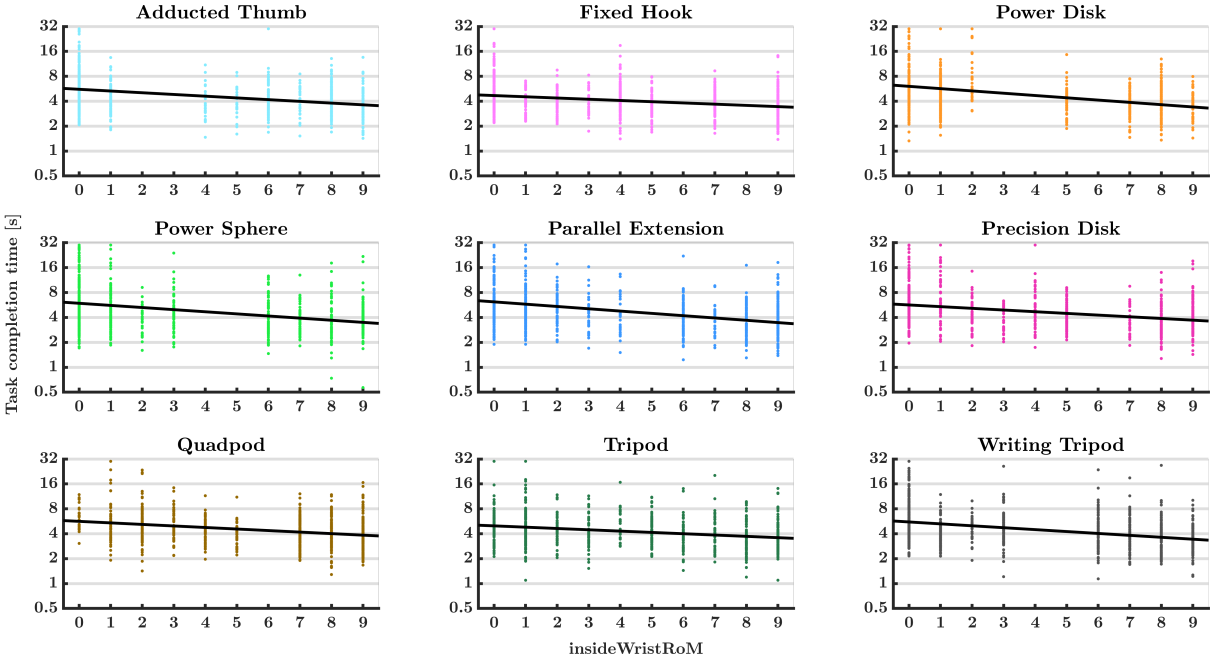

Figure S1. The relationship between the task completion time and *insideWristRoM*, separated by grasp-type handle. *insideWristRoM* designates how many participants reached a specific hole orientation when only movements of the hand and wrist were allowed and is thus a measure for the accessibility of each hole orientation (*insideWristRoM* from 0 to 9, data from Zoller et al. (2019)). The black solid lines depict the resulting linear mixed model with two fixed effects (*handle* and *insideWristRoM*) as well as an interaction of these two fixed effects (Eq. 8 in the main paper). *Note that despite the significant interactions, all the slopes are descending, indicating that pegs were inserted faster in hole locations of more accessible workspace.*

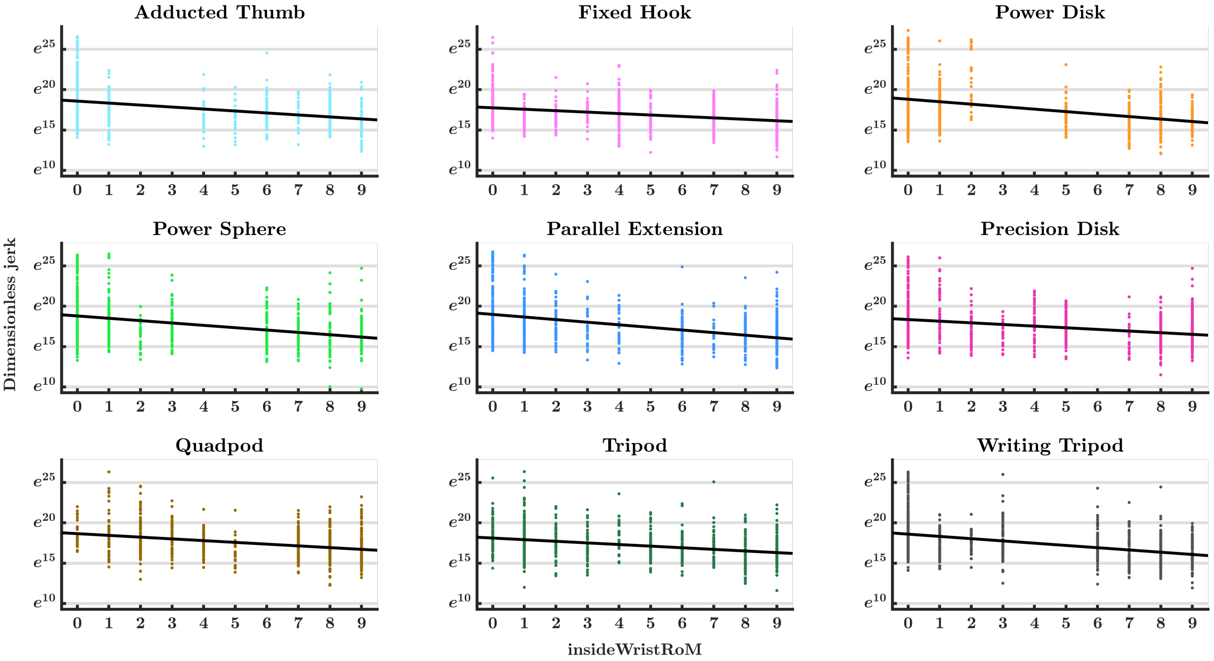

Figure S2. The relationship between the dimensionless jerk and *insideWristRoM*, separated by grasp-type handle. *insideWristRoM* designates how many participants reached a specific hole orientation when only movements of the hand and wrist were allowed and is thus a measure for the accessibility of each hole orientation (*insideWristRoM* from 0 to 9, data from Zoller et al. (2019)). Low y-axis values correlate with high movement smoothness. The black solid lines depict the resulting linear mixed model with two fixed effects (*handle* and *insideWristRoM*) as well as an interaction of these two fixed effects (Eq. 8 in the main paper). *Note that despite the significant interactions, all the slopes are descending, indicating that pegs were inserted with smoother movements in hole locations of more accessible workspace.*

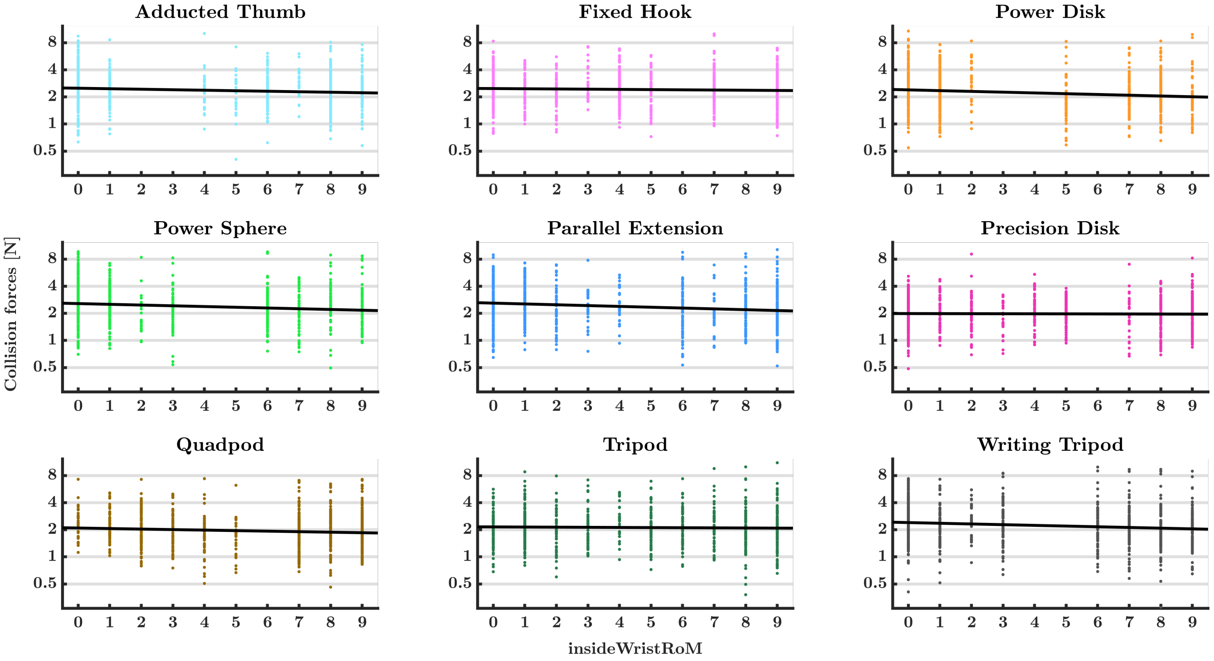

Figure S3. The relationship between the collision forces and *insideWristRoM*, separated by grasp-type handle. *insideWristRoM* designates how many participants reached a specific hole orientation when only movements of the hand and wrist were allowed and is thus a measure for the accessibility of each hole orientation (*insideWristRoM* from 0 to 9, data from Zoller et al. (2019)). The black solid lines depict the resulting linear mixed model with two fixed effects (*handle* and *insideWristRoM*) as well as an interaction of these two fixed effects (Eq. 8 in the main paper). *Note that despite the significant interactions, all the slopes are descending, indicating that pegs were inserted more gentle in hole locations of more accessible workspace.*

4 HISTOGRAMS AND NORMAL Q-Q PLOTS

The histograms of the raw data and, where applicable, the log-transformed raw data are shown in Figs. S4 to S7.

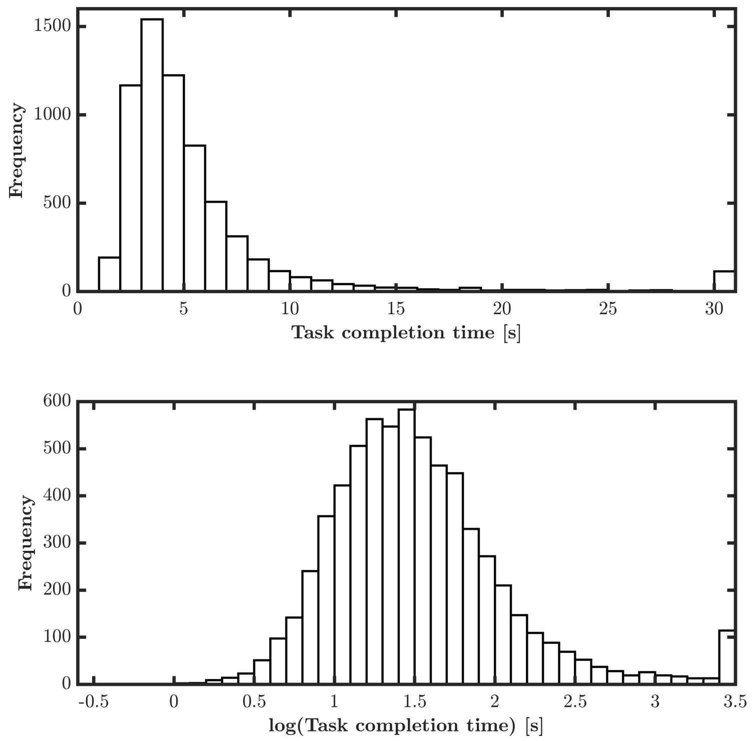

Figure S4. The distribution of the recorded task completion time data and their log-transforms.

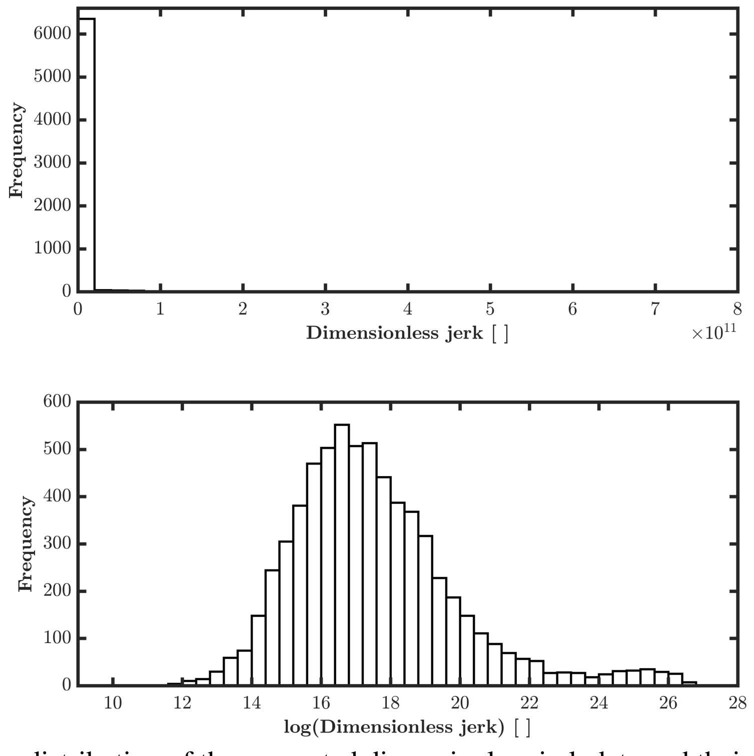

Figure S5. The distribution of the computed dimensionless jerk data and their log-transforms.

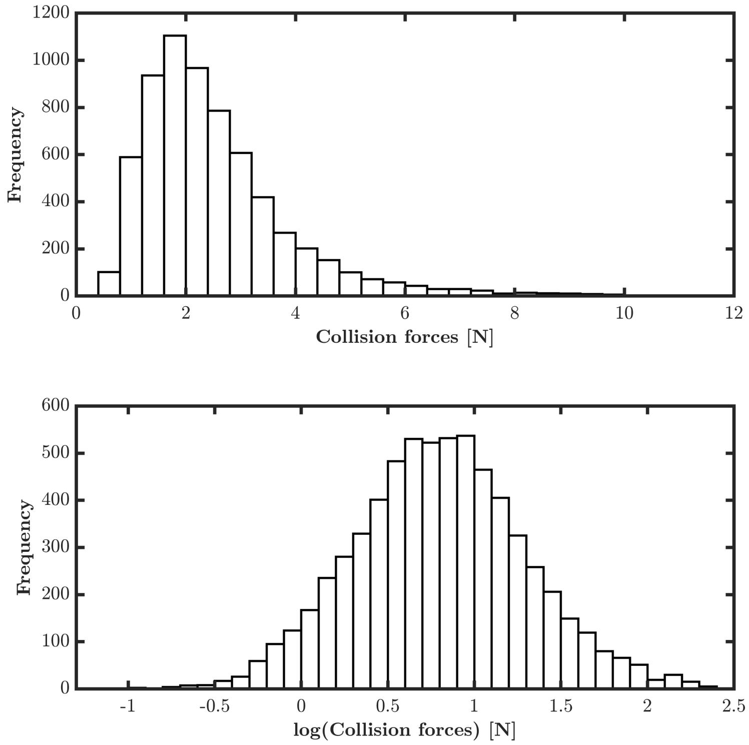

Figure S6. The distribution of the computed collision force data and their log-transforms.

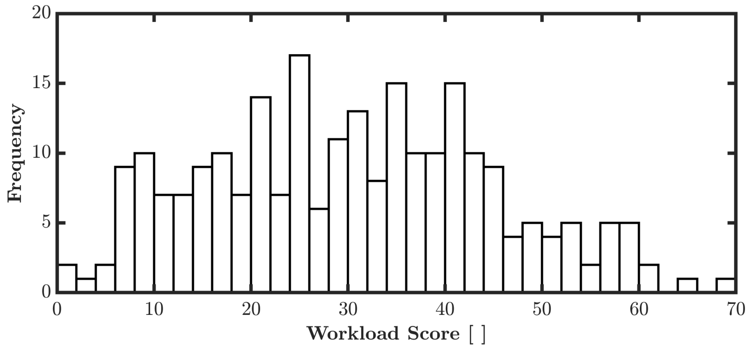

Figure S7. The distribution of the collected workload scores.

The normal Q-Q plots for all linear mixed models (LMMs) resulting from the statistical analysis are shown in Figs. S8 to S11, allowing the reader to assess the assumption for normally distributed residuals.

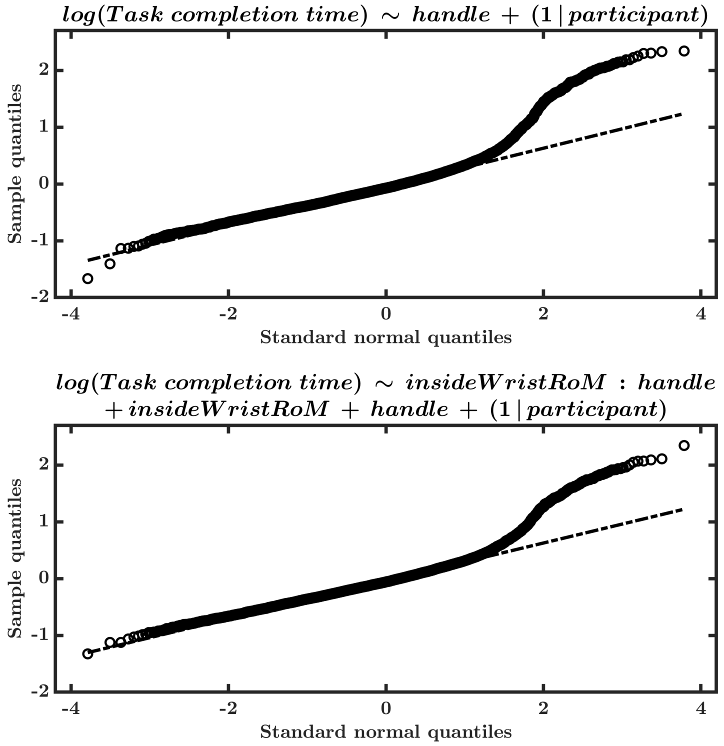

Figure S8. The quantiles of the residuals of the resulting linear mixed models for the task completion times against the theoretical quantile values from a normal distribution. The circles indicate the individual residuals. The solid line joins the first and third quartiles of the residuals, while the dashed line represents the extrapolation of the quartile line, extended to the minimum and maximum values.

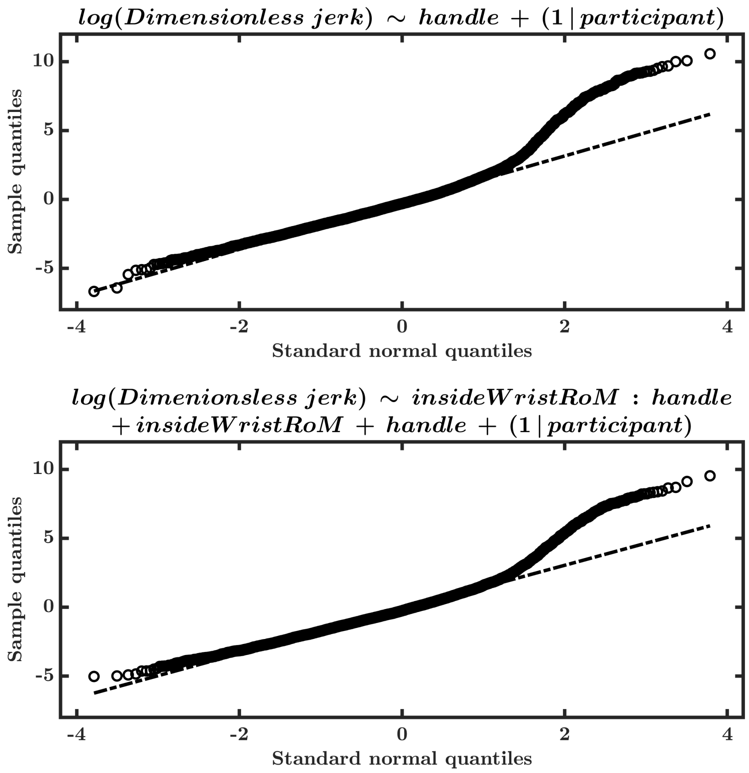

Figure S9. The quantiles of the residuals of the resulting linear mixed models for the dimensionless jerk against the theoretical quantile values from a normal distribution. The circles indicate the individual residuals. The solid line joins the first and third quartiles of the residuals, while the dashed line represents the extrapolation of the quartile line, extended to the minimum and maximum values.

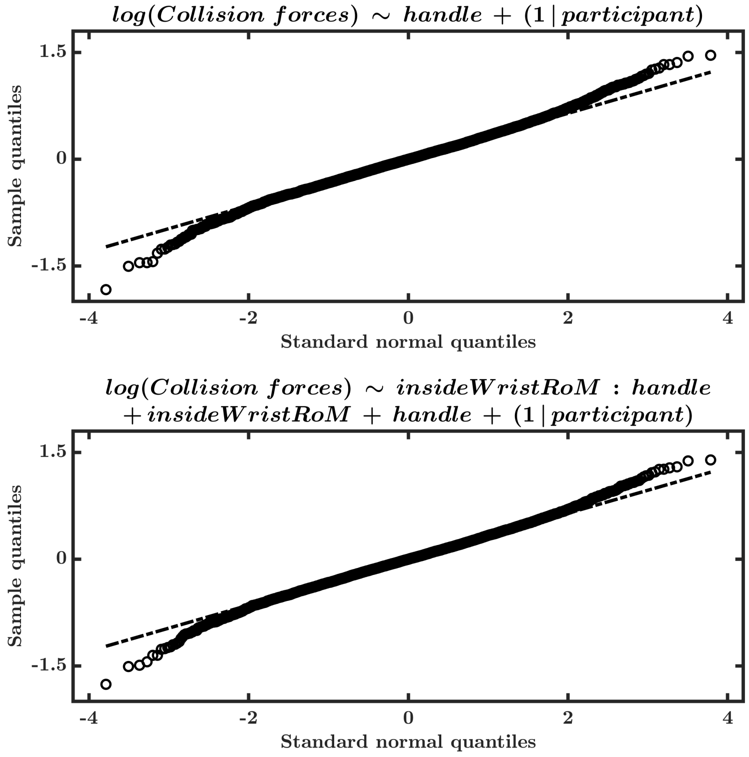

Figure S10. The quantiles of the residuals of the resulting linear mixed models for the collision forces against the theoretical quantile values from a normal distribution. The circles indicate the individual residuals. The solid line joins the first and third quartiles of the residuals, while the dashed line represents the extrapolation of the quartile line, extended to the minimum and maximum values.

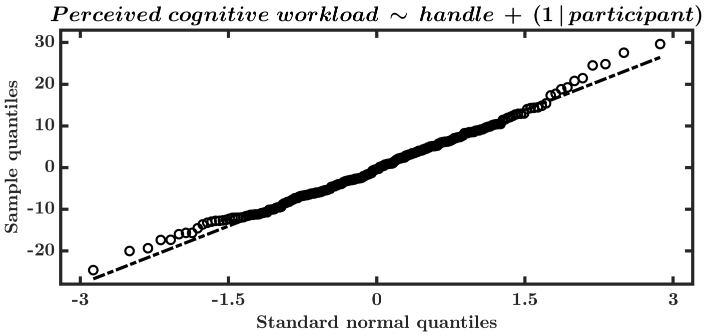

Figure S11. The quantiles of the residuals of the resulting linear mixed model for the perceived workload against the theoretical quantile values from a normal distribution. The circles indicate the individual residuals. The solid line joins the first and third quartiles of the residuals, while the dashed line represents the extrapolation of the quartile line, extended to the minimum and maximum values.

REFERENCES

Byers, J. C., Bittner, A., and Hill, S. G. (1989). Traditional and raw task load index (TLX) correlations: Are paired comparisons necessary. *Advances in Industrial Ergonomics and Safety* 1, 481–485

Zoller, E. I., Cattin, P. C., Zam, A., and Rauter, G. (2019). Assessment of the functional rotational workspace of different grasp type handles for the lambda. 6 haptic device. In *2019 IEEE World Haptics Conference (WHC)* (IEEE), 127–132. doi:10.1109/WHC.2019.8816080
